# Investigating the Efficiency of Hydroxycinnamic Acids to Inhibit the Production of Enniatins by *Fusarium avenaceum* and Modulate the Expression of Enniatins Biosynthetic Genes

**DOI:** 10.3390/toxins12120735

**Published:** 2020-11-24

**Authors:** Charlotte Gautier, Laetitia Pinson-Gadais, Marie-Noelle Verdal-Bonnin, Christine Ducos, Judith Tremblay, Sylvain Chéreau, Vessela Atanasova, Florence Richard-Forget

**Affiliations:** INRAE, Mycology and Food Safety (MycSA), F-33882 Villenave d’Ornon, France; charlotte.gautier@inrae.fr (C.G.); laetitia.pinson-gadais@inrae.fr (L.P.-G.); marie-noelle.verdal-bonnin@inrae.fr (M.-N.V.-B.); christine.ducos@inrae.fr (C.D.); tremblay.jud@hotmail.com (J.T.); sylvain.chereau@inrae.fr (S.C.); vessela.atanasova@inrae.fr (V.A.)

**Keywords:** *Fusarium avenaceum*, enniatins, phenolic acids, transcriptional control

## Abstract

Enniatins (ENNs) that belong to the group of emerging mycotoxins are widespread contaminants of agricultural commodities. There is currently insufficient evidence to rule out health concerns associated with long-term exposure to ENNs and efforts must be strengthened to define a control strategy. While the potential of plant compounds to counteract the contamination with legislated mycotoxins has been reported, little remains known regarding ENNs. The present study evidenced for the first time the efficiency of hydroxycinnamic acids to inhibit the fungal growth and ENNs yield by *Fusarium avenaceum*. Notably, 0.5 mM of exogenous ferulic, caffeic, and *p*-coumaric acids led to a drastic reduction of ENNs synthesis in pH4 broths, with ferulic acid being the most potent. The ENNs production inhibitory activity of ferulic acid was shown to be associated with a significant down-regulation of the expression of ENNs biosynthetic genes. To further investigate the bioactivity of ferulic acid, its metabolic fate was characterized in fungal broths and the capacity of *F. avenaceum* to metabolize it through a C2-cleavage type degradation was demonstrated. Overall, our data support the promising use of ferulic acid in ENNs control strategies, either as part of an environmentally friendly plant-care product or as a biomarker of plant resistance.

## 1. Introduction

Mycotoxins are a critical food safety concern in various agricultural commodities, posing significant health risk to humans and livestock. These fungal metabolites have also a significant economic impact that includes increased health care cost, reduced livestock production, losses in crops, or product recalls. This economic consideration also embraces the costs of mycotoxins management linked to prevention, sampling, analysis, mitigation, and litigation. Extensive research efforts have been made during the past decades with the aim to reduce the levels of mycotoxins in food and feedstuffs [1]. Since mycotoxins are difficult to eliminate once they are present in commodities, prevention methods, gathering pre-harvest field management and post-harvest storage, are acknowledged as the most efficient strategies to ensure food and feed safety and have been one of the major research topics related to mycotoxins [2,3,4].

Currently, more than 400 mycotoxins produced by a variety of fungal species have been evidenced. Among this huge number of metabolites, a small set of approximately 30 mycotoxins, designed as major mycotoxins because of their high toxicological relevance, has been the subject of in-depth studies. These major mycotoxins include *Aspergillus* mycotoxins (aflatoxins and ochratoxins), *Penicillium* mycotoxins (ochratoxins and patulin), and *Fusarium* mycotoxins (deoxynivalenol, zearalenone, fumonisins). These toxins are regulated in many countries. In Europe [5], the regulation (EC) No 1881/2006 established maximum permissible limits for these major mycotoxins in various foodstuffs. In the two past decades, a class of lesser-known mycotoxins, gathered under the term “emerging mycotoxin” has gained increased interest among the scientific community. There is no clear definition for the term “emerging mycotoxin”; it covers a range of mycotoxins that are neither routinely analyzed nor legislatively regulated, the occurrence of which is increasingly documented [6]. The *Fusarium* mycotoxins enniatins (ENNs), beauvericin, moniliformin, and fusaproliferin fall into this group of mycotoxins. The most prevalent toxins among the so-called emerging mycotoxins are ENNs [7], which can be found in a wide range of substrates, including potato, apple, sugar beet, coffee beans, and cereal grains, with cereals being the most commonly contaminated agricultural commodities [8]. The map of ENNs occurrence in European cereal harvests, recently drawn by Gautier et al. [9], clearly illustrates the high frequency of ENNs contamination events together with the high values of contamination levels that can exceed 500 mg · kg^−1^ for certain crops and countries.

**Figure 1:**
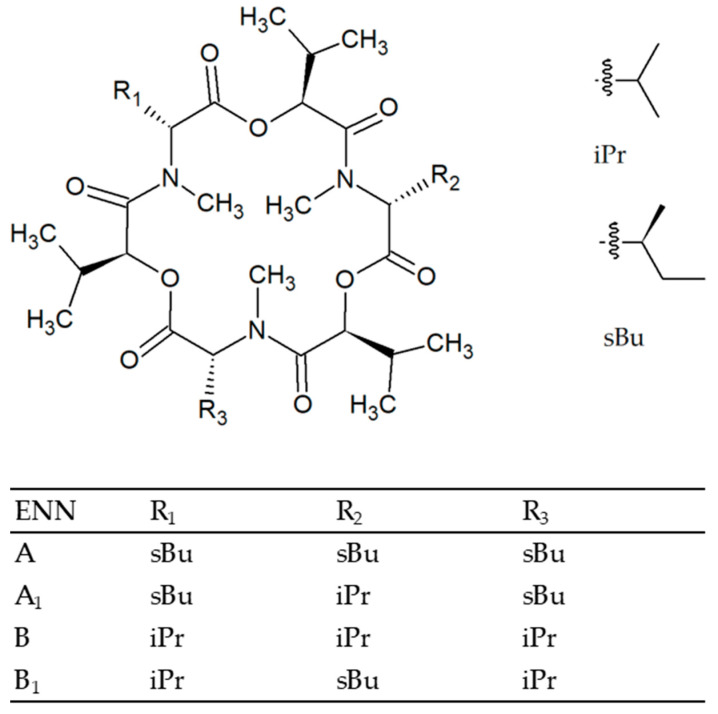
Chemical structure of enniatins A, A_1_, B and B_1_.

ENNs are cyclic hexadepsipeptides. To date, 29 ENNs that are distinct from each other by the nature of the N-methylamino acid residues have been identified [10]. Among these 29 variants, ENNs A, A_1_, B and B_1_ are the most frequently detected in cereal grains; their chemical structures are reported in Figure 1. Nearly 30 fungal species have been shown to have the capacity to yield ENNs, with *Fusarium avenaceum* and *Fusarium tricinctum* being the major contributors to the contamination of cereal grains [9]. While the chemical pathway of ENN biosynthesis has been well documented [11], its genetic basis is less known. In addition to *esyn1* encoding the enniatin synthetase, a non-ribosomal peptide synthetase, that plays a primary role in the biosynthetic pathway of ENNs, only one other gene, *kivr*, that encodes a ketoisovalerate reductase, has been related to ENNs biosynthesis [9]. Notwithstanding the high prevalence of ENNs in food and feed products, sometimes even in high concentrations, there are still significant knowledge gaps regarding their toxicity. Actually, whereas their cytotoxicity has been widely investigated, leading to the demonstration of various effects, including apoptosis, mitochondrial damages, and reactive oxygen species production [12], their in vivo toxicity remains poorly documented. There are not sufficient in vivo toxicity data to allow performing a human risk assessment and not enough evidence to rule out concern about long-term exposure [13]. The issue of interacting toxic effects when ENNs co-occur with other major mycotoxins should also not be neglected [14]. Thus, in the light of the current scientific knowledge, health risks induced by ENNs exposure cannot be excluded and efforts must be pursued to define efficient control strategies to minimize the presence of these mycotoxins in agricultural commodities.

The preharvest strategy package recommended to reduce *Fusarium* mycotoxins in cereal harvests combines several interventions, including the adaptation of the crop sequence, crop residue management, choice of cultivar, proper use of fungicides and appropriate sowing date and rate [15]. In recent years, the environmental and health risks associated with chemical pesticides, plus the risk of increased spreading of *Fusarium* isolates resistant to commonly used synthetic fungicides, have stimulated research into alternative methods of disease management. Prevention measures based on the use of phytochemicals with antimicrobial activities have been investigated [16]. Increasingly, plant phenolic compounds have become the subject of mycotoxin research and many groups have demonstrated their capacity to restrain the fungal growth and the production of various mycotoxins, including *Fusarium* mycotoxins, such as deoxynivalenol [17,18,19], T2 and HT2 toxins [20,21], and fumonisins [22,23]. However, to the best of our knowledge, no study has examined the effect of phenolic compounds on the growth of ENNs-producing *Fusarium* species and on ENN biosynthesis. Among phenolic compounds, hydroxycinnamic acids, that are the most widely distributed phenolic acids in plants, have been frequently reported to display the highest bioactivity against toxigenic fungi [24]. This high efficiency has been ascribed to their lipophilic and antioxidant properties, two characteristics assumed to play a primary role in antifungal and mycotoxin inhibitory activities [25].

The present work aimed at providing first insights on the potential of hydroxycinnamic acids such as caffeic (CAF), ferulic (FER), *p*-coumaric (COUM), chlorogenic (CHLO), sinapic (SIN), and syringic (SYR) acids to interfere with the growth of *F. avenaceum* and its production of ENNs. To a better understanding of the mechanisms involved in the modulation of ENNs yields, the capacity of selected hydroxycinnamic acids to modulate the expression of ENNs biosynthetic genes was analyzed. The study was complemented with further investigation into the way hydroxycinnamic acids can be degraded or converted by *F. avenaceum*.

## 2. Results

### 2.1. Effect of Hydroxycinnamic Acids on the Radial Growth of F. avenaceum I612

The *F. avenaceum* I612 strain was selected as a model strain throughout this study, according to its high toxigenic potential (Table 4, material and methods section) and capacity to produce ENNs over a wide range of pH conditions. Concentrations that inhibit 50% of the radial growth (IC_50_ values) of *F. avenaceum* I612 cultivated on solid potato dextrose agar (PDA) medium were determined for six selected hydroxycinnamic acids and are gathered in Table 1. The determined IC_50_ values ranged from 1.4 mM to 5.2 mM. The lower the IC_50_ value, the higher the fungicidal efficiency of the phenolic acid under consideration. Thus, FER and COUM were characterized as the most toxic compounds, whereas CAF and CHLO displayed the weakest antifungal effect against *F. avenaceum*.

### 2.2. Effect of Hydroxycinnamic Acids on Mycelium Weights and Production of ENNs by F. avenaceum I612

The antifungal effect and mycotoxin inhibitory activity of the six selected hydroxycinnamic acids were further investigated in buffered liquid media inoculated with *F. avenaceum* I612. A concentration of 0.5 mM, which is close to the amounts of phenolic acids in wheat grains [26], was chosen. Two pH conditions (pH 3 and pH 6) were considered to flank the pKa values of the phenolic acids and evaluate the contribution of dissociated and non-dissociated forms to their bioactivity. pKa values of the studied phenolic acids, mycelium weights, and ENNs amounts are gathered in Table 2. Regarding mycelium weight data, our results indicated the occurrence of a substantial inhibition of *F. avenaceum* fungal biomass accumulation (although not statistically significant) for three conditions: cultures at pH 3 supplemented with 0.5 mM FER, SIN, and SYR acids. In 10-day-old cultures, mycelium weights were reduced by 43%, 36% and 31% in presence of FER, SIN, and SYR, respectively. A weak (10%) but statistically significant inhibition was also noticed for CAF at pH 6 while a statistically significant increase in fungal biomass was induced by exposure to CAF and CHLO at pH 3 and FER at pH 6. A comparative analysis of the amounts of ENNs (sum of ENN A, A_1_, B and B_1_) produced by *F. avenaceum* I612 in pH 3 and pH 6 buffered liquid cultures supplemented or not with hydroxycinnamic acids (0.5 mM) revealed that, in pH 3 media, a significant inhibition of the ENN biosynthesis was induced by phenolic acid supplementation, regardless of the hydroxycinnamic acid. Notably, no ENNs or only traces of ENNs were detected in CAF, FER and COUM treated broths. A drastic reduction in ENNs yields was also observed in presence of CHLO, SIN and SYR acids with inhibitory ratios reaching 98%, 92%, and 80%, respectively. In pH 6 modalities, for which dissociated forms of hydroxycinnamic acids are predominant, a statistically significant reduction of ENNs production was evidenced for FER and COUM treatments. The inhibition percentages were lower than those quantified in media buffered at pH 3, close to 60% (FER) and 70% (COUM). No inhibition was observed with SIN, SYR and CHLO. The reduction induced by CAF was not statistically significant.

### 2.3. Effect of FER, CAF and COUM on Fungal Biomass and Production of ENNs by a Panel of F. avenaceum Strains

**Figure 2:**
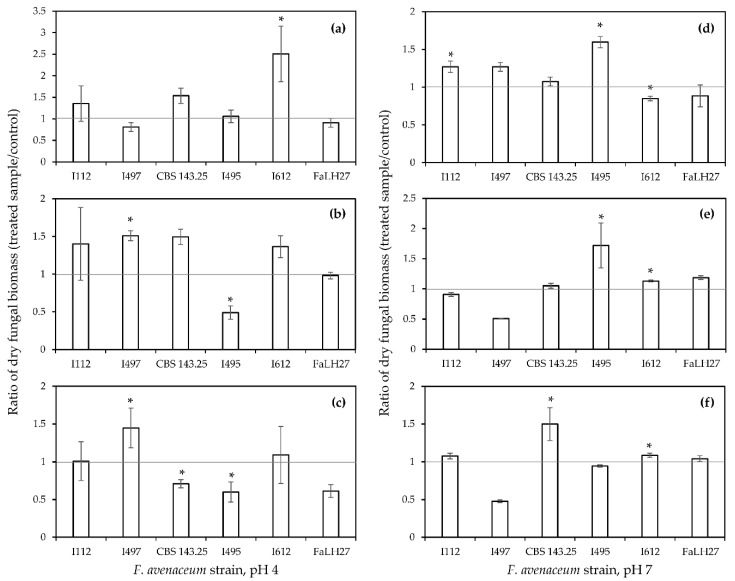
Effect of (**a**,**d**) caffeic acid, (**b**,**e**) *p*-coumaric acid and (**c**,**f**) ferulic acid at 0.5 mM on the fungal biomass weights of six *F. avenaceum* strains in 10-day-old broths buffered at (**a**–**c**) pH 4 and at (**d**–**f**) pH 7. Data are means ± standard deviation using three biological replicates. Differences between control and phenolic acid-treated cultures were determined with multiple comparisons using Tukey test. Asterix (*) indicates significant differences when compared with corresponding controls (*p* < 0.05).

**Figure 3:**
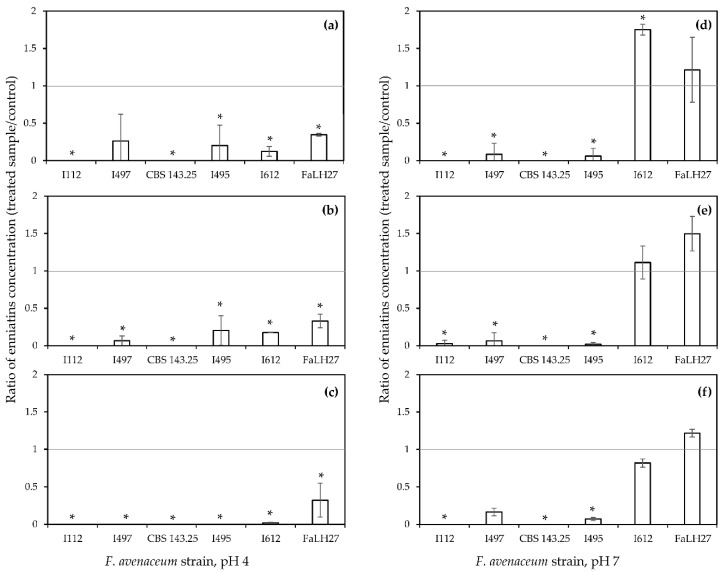
Effect of (**a**,**d**) caffeic acid, (**b**,**e**) *p*-coumaric acid and (**c**,**f)** ferulic acid at 0.5 mM on enniatins production by six *F. avenaceum* strains in 10-day-old broths buffered at (**a**–**c**) pH 4 and at (**d**–**f**) pH 7. Data are means ± standard deviation using three biological replicates. Differences between control and phenolic acid-treated cultures were determined with multiple comparisons using Tukey test. Asterix (*) indicates significant differences when compared with corresponding control (*p* < 0.05).

The effect of 0.5 mM CAF, COUM, and FER—the three phenolic acids characterized by the highest bioactivity in the previous trial—was further evaluated using five additional *F. avenaceum* strains with different capacities to produce ENNs (Table 4, material and methods section). Two strains of the panel were low-producing ones (I112 and CBS 143.25) and three were high-producing isolates (I495, I497, and FaLH27) characterized by ENNs yields close or higher than 1000 µg · g^−1^ of dried mycelium in 10-day-old broths. The I612 strain belongs to this group of high-producing isolates. pH values of the liquid buffered media were set at pH 4 and pH 7 to flank pKa values of the phenolic acids whereas allowing the production of ENNs by each of the considered strains in all untreated cultures. Results (mycelium weights and ENNs) are expressed as the ratio between the amounts quantified in the treated cultures and in control ones (Figure 2 and Figure 3).

Regarding fungal growth impact in pH 4 buffered media (Figure 2a–c), a significant reduction in fungal biomass was observed for two strains in FER-treated cultures (I495 and CBS 143.25) with inhibition rates ranging from 25% to 55%, for one strain with COUM (I495—inhibition rate close to 50%) whereas no significant inhibition was evidenced in cultures supplemented with CAF. In pH 7 buffered media, a substantial inhibition (close to 50%) of the fungal growth was observed for two modalities: I497 broths supplemented with COUM and FER. It should also be noticed that for some treatments and strains, a statistically significant increase in fungal biomass was induced by 0.5 mM of exogeneous phenolic acid. Such an increase was observed in CAF-treated cultures for I612 at pH 4 and I112 and I495 at pH 7, in COUM-treated cultures for I497 at pH 4 and I495 and I612 at pH 7, in FER-treated cultures for I497 at pH 4 and CBS 143.25 and I612 at pH 7. Altogether, our data clearly evidence the highly variable sensitivity of *F. avenaceum* strains to the three selected phenolic acids. According to the number of strains with a reduced growth, FER appears to be a more potent antifungal compound than COUM and CAF.

Looking at ENNs yields, results reported in Figure 3 clearly evidence the drastic inhibition induced by each of the three phenolic acids in pH 4 buffered media, regardless of the inoculated isolate. Notably, exogenous FER abrogated the production of ENNs for four strains (I112, I495, I497 and CBS 143.25), led to traces of ENNs in I612 broths and to a 65% reduction of the ENNs amounts produced by the FaLH27 isolate. In pH 7 buffered media, with the exception of two strains, I612 and FaLH27, for which the production of ENNs was not affected, increased (I612 in CAF-treated cultures and FaLH27 in COUM-treated cultures) or weakly inhibited (I612 in FER-treated cultures), supplementation with CAF, COUM and FER led to a substantial reduction in ENNs amounts.

Taken together, the previous data suggest that the effects on fungal growth and on ENNs biosynthesis induced by hydroxycinnamic acids can be two independent events. Indeed, for most of the modalities, a significant reduction in ENNs yields was observed while the fungal growth was not affected. Moreover, even though inhibitory effects on fungal growth and ENNs yields were shown in pH 7 buffered media, the number of affected strains and the inhibition rates were broadly higher in pH 4 conditions. These data corroborate the highest bioactivity of non-dissociated forms of phenolic acids.

### 2.4. Effect of FER on the Expression of ENNs Biosynthetic Genes from F. avenaceum I612

**Figure 4:**
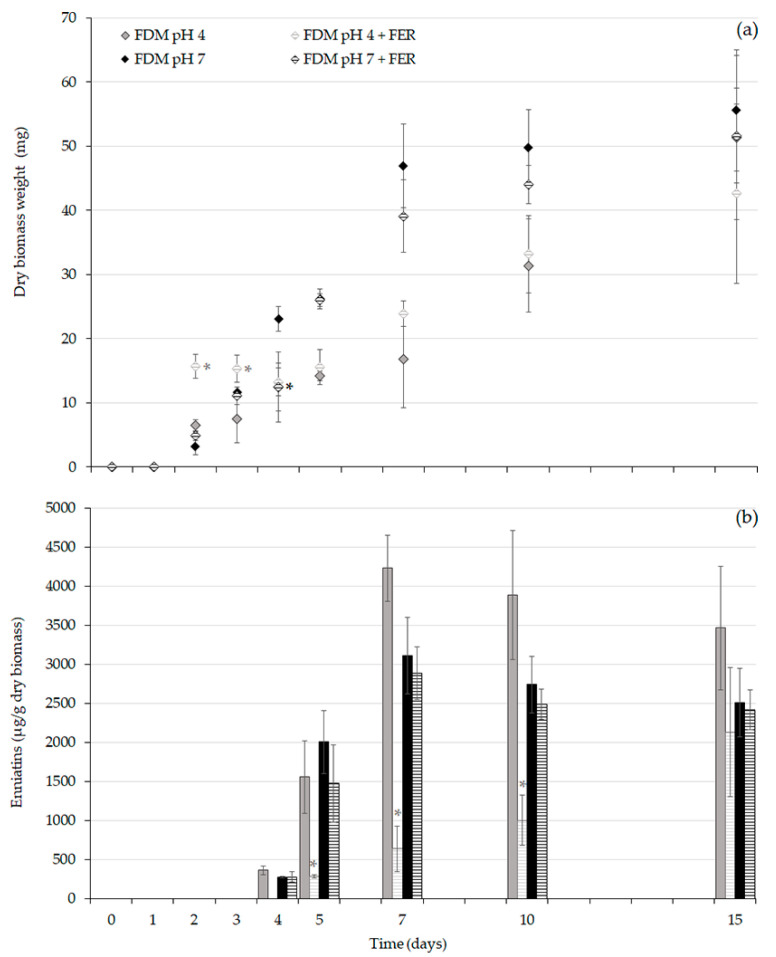
Kinetics of (**a**) fungal growth and (**b**) enniatins production by *F. avenaceum* I612 in *Fusarium* defined media buffered at pH 4 (grey) and at pH 7 (black) supplemented (striped grey or striped black) or not (full grey or full black) with ferulic acid at 0.5 mM. Data are means ± standard deviation using three biological replicates. Differences between control and ferulic acid-treated cultures in terms of fungal biomass and enniatins were determined separately for each day with multiple comparisons using Tukey test. Asterix (*) indicates significant differences when compared with corresponding control (*p* < 0.05). FER—ferulic acid, FDM—*Fusarium* defined medium.

To further investigate the mechanism by which FER can affect the production of ENNs, the expression of two genes involved in the ENNs biosynthesis pathway (*esyn1* and *kivr*) was analyzed. The expression of *esyn1* and *kivr* was compared from *F. avenaceum* I612 strain grown in pH 4 and pH 7 media supplemented or not with 0.5 mM FER. As reported on Figure 4, the production of ENNs in the culture media used for this study starts at day 4, speeds up between day 4 and day 7, then stabilizes. We investigated gene expression levels at day 5, i.e., when ENNS accumulation is rapidly increasing. Levels of the elongation factor *ef1α* and the RNA polymerase II *(rpb2)* genes, used as reference genes, were not different between ferulic-treated cultures and untreated cultures. The expression values of the target *esyn1* and *kivr* genes were normalized by the reference gene expression values and were expressed as log2 fold change values in the treated conditions relative to the control conditions (Table 3).

Our data indicate a substantial downregulation of the expression of *esyn1* and *kivr* induced by exogenous FER in the pH 4 medium. Indeed, the log2 fold inhibition values reached −11.5 for *esyn1* and −8.4 for *kivr*, meaning that the *esyn1* transcript abundance was reduced by more than 2900 times and that of *kivr* by more than 330 times. This result suggests that the decrease in ENNs production observed in pH 4 FER-treated cultures (as clearly seen in Figure 4b) could be ascribed to a decrease in the level of biosynthetic genes expression. In pH 7 media, the expression of *esyn1* and *kivr* was minimally affected by the FER treatment (log2 change values were –0.75 for *esyn1* −0.6 for *kivr*), in accordance with the lack of observed effect on toxin production (Figure 4b). In unbuffered FDM cultures for which the fungal growth is accompanied by a pH increase (from 4.3 at day 0 to 6 at day 5), FER supplementation led to a reduction of *esyn1* and *kivr* transcript abundance by a factor close to 10, in accordance with the weak reduction (< 20%) of ENNs production observed at day 5 (data not shown).

### 2.5. Biotransformation of FER in F. avenaceum I612 Broths

**Figure 5:**
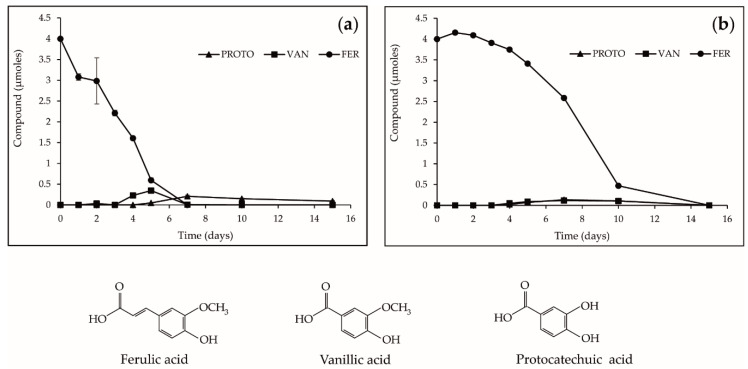
Time-course of ferulic acid decline in *F. avenaceum* I612 cultures in *Fusarium* defined medium buffered at (**a**) pH 4 and at (**b**) pH 7. Chemical structure of ferulic acid and its metabolites. Values are expressed as means ± standard deviation using three biological replicates. FER—ferulic acid, VAN—vanillic acid and PROTO—protocatechuic acid.

To investigate the metabolization pathway of FER by *F. avenaceum*, levels of FER amounts were monitored in culture supernatants over the course of *F. avenaceum* I612 growth in pH 4 and pH 7 media. Results are gathered in Figure 5a,b. First analyses showed that FER levels were stable in pH 4 and pH 7 non-inoculated cultures during the 15 days of incubation at 25 °C (data not shown). In supernatants of pH 4 inoculated cultures (Figure 5a), a rapid decrease in FER amounts during the first days of culture, i.e., during the exponential phase of fungal growth (Figure 4a), was observed, resulting in non-quantifiable levels at day 7. In liquid chromatography/diode-array detector (LC/DAD) profiles of the supernatants monitored at 260 nm, the presence of two additional peaks was highlighted. On the basis of retention times and UV spectra of reference standards, these peaks were identified as vanillic acid (VAN) and protocatechuic acid (PROTO). VAN was only quantified at day 4 and day 5; the highest quantified amount was close to 0.3 µmoles. PROTO was detected starting 5 days after inoculation with levels ranging from 0.1 to 0.25 µmoles. In supernatants of pH 7 inoculated cultures (Figure 5b), the decrease in FER amounts was slower than that observed in pH 4 broths; 12% of the supplemented FER was still present in 10-day-old broths. Similar to what was observed in pH 4 broths, the FER decrease was accompanied by the formation of VAN and PROTO. Regardless of the pH of the media, VAN and PROTO were not present in non-inoculated supernatants supplemented with FER, suggesting that their production could be related to the metabolization of FER by *F. avenaceum*. The unbalanced ratio between FER disappearance (4 µmoles) and VAN + PROTO acids accumulation (below 1 µmole) may indicate the occurrence of additional mechanisms underpinning the decrease in FER. In addition to other metabolization pathways leading to products that were not visualized with the LC/DAD method used throughout this study, FER disappearance could also result from its penetration into fungal cells.

## 3. Discussion

According to the increasing evidence of the worldwide and frequent occurrence of crop contamination with ENNs, together with the potential risk of health hazards associated with chronic exposure and the likelihood of synergistic toxic effects when ENNs co-occur with other regulated mycotoxins, ENNs might pose a major threat to human and animal health [9]. Efforts should be pursued to define efficient control strategies to mitigate their presence in cereal harvests. Among pre- and post-harvest measures commonly applied to combat toxigenic fungi and minimize the contamination with mycotoxins, chemical control is widely used. In recent years, accelerated efforts have been undertaken in the screening of plant metabolites for their potential use as alternatives to synthetic fungicides and the development of environmental-friendly solutions to counteract the growth of toxigenic fungi and their production of mycotoxins [16]. These efforts have been mainly concentrated on legislated mycotoxins, intensely on aflatoxins, ochratoxins, trichothecenes, and fumonisins [27,28]. The present study evidenced for the first time the capacity of several plant hydroxycinnamic acids to inhibit the fungal growth of ENNs producing fungal isolates, and to efficiently reduce the production of ENNs. Notably, exogenous FER, CAF, and COUM were shown to substantially reduce the production of ENNs by a set of *F. avenaceum* strains cultivated at pH 4. Taken together, our data indicated that, among the most abundant hydroxycinnamic acids occurring in plant tissues, FER possessed the highest fungicidal activity against *F. avenaceum* but also the highest ENNs inhibitory activity. Notably, a FER treatment synchronized with spore inoculation was shown to drastically reduce the amounts of ENNs in 10-day old broths, evidencing a lasting inhibitory effect. Such a lasting effect could be explained by the strong imprint of germination process on the biosynthesis of mycotoxins [17,29]. Our findings corroborate previous studies that have led to the conclusion that FER was the most potent phenolic acid with antifungal and anti-mycotoxin activity against various *Fusarium* species including *Fusarium graminearum*, *Fusarium verticillioides*, *Fusarium poae*, *Fusarium langsethiae*, and *Fusarium sporotrichioides* [20,25,30]. According to our results, FER that has been suggested to play a pivotal role in cereal resistance to *F. graminearum* and to deoxynivalenol accumulation [24,31] could also contribute to the mechanisms employed by plants to counteract the infection by *F. avenaceum* and contamination with ENNs. However, our data have also indicated that, in certain situations, exposure to sublethal concentrations of FER, CAF and COUM can result in an increased biomass. Such increasing growth effects induced by phenolic acid exposure have previously been reported in the literature. Ferruz et al. [20] have shown that the growth of *F. langsethiae* was enhanced with 0.5 mM of FER while 1 mM of FER led to a reduction of the fungal biomass. Likewise, Schöneberg et al. [21] have reported that the growth of *F. graminearum* and *F. poae* was increased by exposure to *p*-hydroxybenzoic acid. According to Kulik et al. [19], some phenolic acids could trigger a stimulation of the biosynthesis of ergosterol in *F. graminearum* and *F. culmorum*. An upregulation of ergosterol biosynthetic genes upon phenolic exposure has also been reported to explain the tolerance of *Saccharomyces cerevisiae* [32]. To try deciphering the origin of the growth increasing effects that we have observed in the present study, it would be worth investigating the impact of FER, CAF, and COUM exposure on the biosynthesis of ergosterol in *F. avenaceum*.

Our data have shown that lowering the pH value of the culture media led to an increased bioactivity of hydroxycinnamic acids. A similar trend has been reported regarding the antimicrobial properties of various phenolic acids, including hydroxycinnamic and benzoic acids [33,34]. Actually, the concentration of undissociated forms of phenolic acids increases with decreasing pH and these undissociated forms are more lipophilic compared to dissociated phenolic acids. Undissociated forms are therefore more prone to penetrate the fungal membrane and to induce irreversible perturbations including hydrophobicity modifications, changes in negative surface charge and the formation of pores leading to leakage of essential intracellular constituents [35]. The faster disappearance of FER observed during the exponential phase of mycelial growth of *F. avenaceum* cultivated in pH 4 media compared to pH 7 culture media (Figure 4) is consistent with the highest solubility of undissociated forms in the fungal membrane. Moreover, our results suggested that FER disappearance could also result from its biotransformation by *F. avenaceum*. In fact, the analysis of *F. avenaceum* broths showed that the decrease in FER was accompanied by the formation of VAN and PROTO (Figure 5). Bioconversion of FER into VAN by filamentous fungi has been widely studied and different metabolic pathways have been proposed. A first decarboxylation of FER into 4-vinylguaicol followed by its conversion into vanillin and finally the reduction of vanillin into VAN was suggested for *Schizophyllum commune* [36]. For *Pycnoporus cinnabarinus* [37], *Aspergillus niger* and *Phanerochaete crysosporum* [38], the proposed metabolic route includes a first oxidative C2-cleavage, i.e., cleavage of the propenoic side chain of FER, to yield VAN, that can be further oxidized into vanillin or decarboxylated into methoxyquinone. According to our results, neither vanillin nor methoxyquinone were detected in FER-supplemented media inoculated by *F. avenaceum*. We however detected the presence of PROTO which could be explained by the metabolisation of VAN catalyzed by a vanillate-O-demethylase [39,40]. In our experiments, while more than 4 µmoles of FER have disappeared from the culture broths after 7 days of incubation at pH 4 or 10 days at pH 7, the sum of quantified VAN + PROTO acids did not exceed 1 µmole. Even though the occurrence of additional biotransformation pathways of FER, which the chromatographic methods we used did not allow to evidence, cannot be excluded, these findings could suggest that disappearance of FER in *F. avenaceum* broths is mostly due to its integration within the fungal membranes. The weak capacity of *F. avenaceum* to metabolize FER, as suggested by our results, could explain the significant toxic effects of this phenolic acid. According to the publication of Shalaby et al. [41] focusing of another fungal plant pathogen, *Cochliobolus heterostrophus*, two classes of phenolic hydroxycinnamic acids could be distinguished according to their antifungal effects. One class (to which FER belongs) gathers phenolic acids that are very little metabolized by the fungus and are highly toxic. A second class (including CAF and COUM) gathers phenolic acids that have the capacity to induce the expression of fungal genes encoding phenolic acid degrading enzymes and are consequently less toxic. Our results, that have shown the higher toxicity of FER compared to that of CAF and COUM, are in accordance with the previous classification.

When comparing the fungicidal and anti-mycotoxin activities of FER, CAF and COUM acids (Figure 2 and Figure 3), it appeared that these two effects could be related to independent mechanisms. Indeed, for several modalities, a significant reduction in ENNs yield was observed, while *F. avenaceum* growth was not or weakly affected. As clearly demonstrated by our results, exogenous FER induced a drastic decrease of the expression of ENNs biosynthetic genes, which corroborates the occurrence of a regulatory mechanism that specifically targets the production of ENNs. These data are in accordance with previously published reports that have indicated that the efficiency of some natural plant compounds to inhibit the production of mycotoxins was associated with a transcriptional down-regulation of biosynthetic genes. For instance, the expression of key genes involved in the biosynthesis of aflatoxin B1 and ochratoxin A was shown to be significantly reduced by exposure to cinnamaldehyde, a natural plant substance derived from cinnamon [42,43]. In the same way, the inhibition of the biosynthesis of type B (deoxynivalenol) and type A (toxins T-2 and HT-2) trichothecenes by FER has been partially ascribed to a decrease in the expression of several *tri* genes [17,20]. Interestingly, the intensity of the reduction of the expression of ENNs biosynthetic genes induced by exogenous FER was remarkably higher than that observed in the studies mentioned above; regulation factors higher than 2900 for *esyn1* and 330 for *kirv* were quantified in the present study while these factors were lower than 10 regarding trichothecenes biosynthetic genes [17,20]. The mechanism that can explain the high efficiency of FER to modulate the expression of *esyn1* needs to be elucidated. One hypothesis could be linked to the capacity of FER to alleviate oxidative stress which has been shown to activate the biosynthesis of various mycotoxins and increase the expression of biosynthetic genes [44,45]. From a mechanistic perspective, several reports have evidenced that transcription factors homologs of Yap1 in *S. cerevisiae* played a key role in fungal tolerance to oxidative stress and promoted the production of toxins [46,47]. However, while the connection between oxidative stress response and the regulation of mycotoxin biosynthetic pathway has been investigated for most of the major legislated mycotoxins, nothing is known regarding the effect of oxidative stress on the production of ENNs.

## 4. Conclusions

The results obtained in this study highlight the high efficiency of FER to inhibit the production of ENNs by *F. avenaceum* associated with a notable capacity of this phenolic acid to induce a down-expression of ENNs biosynthetic genes. Our data support the promising use of this natural plant compound in strategies aiming to prevent the contamination of crops with ENNs, either as part of an environmentally friendly plant-care product or as a biomarker of plant resistance. The present study stresses the fact that efforts must be pursued to fully understand the mechanistic clues underpinning the effect of FER on the biosynthesis of ENNs, but also, more generally, the effect of environmental factors on the production of this family of so-called emerging mycotoxins.

## 5. Materials and Methods

### 5.1. Chemicals and Standards

For LC/DAD analysis, LC-grade acetonitrile and methanol were purchased from VWR (Fontenay-Sous-Bois, France). Vanillic, protocatechuic, caffeic, ferulic, *p*-coumaric, sinapic, syringic, and chlorogenic acids, as well as enniatin A, enniatin A_1_, enniatin B, and enniatin B_1_ were purchased from Sigma-Aldrich (Saint-Quentin-Fallavier, France). Reagents were purchased from Scharlau (Barcelona, Spain), Sigma Aldrich (Saint-Quentin-Fallavier, France) and Thermo Fischer Scientific (Illkirch, France).

### 5.2. F. avenaceum Strains, Media and Culture Conditions

Six *F. avenaceum* strains were used in this study; their characteristics and toxigenic potential are summarized in Table 4. They have been assigned as belonging to the *F. avenaceum* species by using qPCR, with species-specific primers [48,49]. These *Fusarium* strains were chosen according to the amount of enniatins they can produce: low producing (I112 and CBS 143.25) and high producing strains (FaLH27, I612, I497, and I495).

Stock cultures were maintained at 4 °C on PDA slants under mineral oil. When inoculum was required, the *Fusarium* strains were grown on PDA plates at 25 °C in the dark for 5 days, and spore suspensions prepared in carboxymethyl cellulose (CMC) liquid medium (15 g CMC, 1 g Yeast extract, 0.5 MgSO_4_, 7 H_2_0, 1 g NH_4_NO_3_, 1 g KH_2_PO_4_, for 1 L medium) and incubated in darkness at 25 °C and at 180 rpm in a Multitron incubator shaker (INFORS AG, Bottmingen, Switzerland). After 3 days, the conidia were recovered using centrifugation (2300× *g* for 10 min), counted in a Thoma chamber and diluted with sterile water to a final concentration of 10^4^ spores · mL^−1^.

Radial growth inhibition was performed on PDA medium in Petri dishes (diameter 90 mm) inoculated at the center with ten microliters of I612 spore suspension at 10^4^ spores · mL^−1^. PDA media were supplemented with CAF, FER, COUM, CHLO, SIN, and SYR at two, three, four, five or six mmol · L^−1^. For each condition, plates (in triplicate) were incubated at 25 °C for six days. Then, 6-day-old plates were photographed, and fungal surface areas were determined using the ImageJ software package (developed by the National Institutes of Health, Bethesda, MD, USA). Growth inhibition values (GIV) were expressed as the ratio [(average surface areas in the control dishes minus the average surface areas in the treated dishes) on the average surface areas in the control dishes]. For each phenolic acid, IC_50_ were determined using the three replicates of the five tested concentrations and dose effect logistic model (XLSTAT software; Addinsoft, Rennes, France).

Liquid culture experiments were performed in FDM medium [50]. Buffered FDM media were prepared using McIlvaine buffer solutions [51]. FDM and buffered FDM were supplemented or not with phenolic acids at 0.5 mM. For each condition, cultures were made in triplicate. Sterile Petri dishes (diameter 55 mm) containing 8 mL of liquid medium were inoculated with 100 µL of spore suspensions at 10^4^ spores · mL^−1^. Fungal liquid cultures were incubated at 25 °C in the dark for 10 days. Additional incubation times were included for the kinetic study (1, 2, 3, 4, 5, 7, 10, and 15 days). After incubation, the cultures were centrifuged at 4 °C and at 5000× *g* for 10 min. The supernatants were stored at −20 °C until analysis. Fungal biomass was measured by weighing the mycelial pellet after 48 h of freeze-drying (Cryotec^®^, Saint Gely du Fesc, France). For gene expression study at day 5, lyophilized mycelia were stored at −80 °C before RNA extraction. Three replications and appropriate controls were carried out for each experimentation. For each trial, it was ensured that supplementation with phenolic acids did not modify the pH values of the treated cultures compared to controls.

### 5.3. Extraction and Analysis of Enniatins

Five milliliters of culture medium were extracted with 10 mL of ethyl acetate. Five milliliters of the organic phase were evaporated to dryness at 45 °C under nitrogen flow. The dried samples were dissolved in 200 μL of methanol/water (1:1, *v*/*v*) and filtered on 0.2 µm filters before LC/DAD analysis. Quantification of A, A_1_, B and B_1_ enniatins was performed on a Shimadzu Prominence UPLC chain, equipped with two pumps LC-30 AD, a degasser DGU-20A5R, an auto sampler SIL-30 AC and a DAD detector SPD-M20A (Shimadzu Scientific Instruments, Noisiel, France). Separation was achieved on a Kinetex 2,6U XB-C18—100 Å column (150 × 4.6 mm; 2.6 µm) (Phenomenex, Le Pecq, France) maintained at 45 °C. The mobile phase consisted of water (solvent A) and acetonitrile (solvent B). The following gradient was used for elution: 30% B for 2.5 min, 30–99% B in 5 min, 99% B for 3.5 min, 99–30% B in 0.5 min, and 2.5 min post-run equilibration with initial conditions. The flow was kept at 1.4 mL · min^−1^. The injection volume was 5 μL. UV-visible spectra were recorded from 190 to 450 nm and peak areas were measured at 205 nm (2.5–50 µg · mL^−1^). Quantification was performed using external calibration with standard solutions. The limit of detection was 0.01 µg · g^−1^. The retention time of ENNs were 8 min for B, 8.2 min for B_1_, 8.4 min for A_1_, and 8.6 min for A.

### 5.4. Extraction and Analysis of Phenolic Acids and Their Transformation Products by I612

Phenolic acids in supernatants of I612 broths were directly analyzed by LC/DAD with a 1100 Series HPLC system (Agilent, France), following the procedure described by Gauthier et al. [18].

### 5.5. Extraction of Total RNA, Preparation of cDNA and Real-Time RT-PCR Analysis

Total RNA was extracted from 5-day-old mycelia of *F. avenaceum* I612 cultivated in pH 4 and pH 7 buffered FDM media supplemented or not with 0.5 mM FER. Three biological replicates were prepared for each condition. Frozen mycelia (50 mg) were ground (Precellys Evolution & Cryolis, Montigny Le Bretonneux, France) with glass beads in 350 µL of lysis buffer (NucleoMag RNA^®^ 96 Kit, Macherey-Nagel, Düren, Germany). Total RNA (separated from the beads and cell debris) was extracted using a MagMAX™ automated system (Thermo Fischer Scientific, Vantaa, Finland) and the NucleoMagRNA^®^ 96 kit (Thermo Fischer Scientific, Vantaa, Finland) including a DNAse treatment following the instructions of the manufacturer. The quality of the prepared RNA was evaluated by agarose gel electrophoresis. The RNA was quantified by spectrometry (Nanodrop 1000/2000, Wilmington, NC, USA). The absence of contamination with genomic DNA was verified by qPCR (Light cycler 2.0, Roche, Risch-Rotkreuz, Switzerland) on 1 µL total RNA (20 ng) mixed with 9 µL of reaction mix using the QuantiFast™ SYBR^®^ Green PCR kit (Qiagen, Courtaboeuf, France).

Reverse transcription was performed with the SuperSript™ IV First-Strand Synthesis System kit (Invitrogen, Vilnius, Lithuania) according to the supplier’s protocol. The cDNA samples were stored at −20 °C until their use for PCR analysis. qPCR analyses were performed with a QuantStudio5™ (ThermoFisher Scientific, Vantaa, Finland) using 1 µL of cDNA (10 ng of reverse transcribed RNA) mixed with 9 µL of reaction mix prepared using the Power Track SYBR^®^ Green Master Mix (Applied Biosystems, Vilnius, Lithuania). The forward and reverse primers used for the genes targeted in the study are reported in Table 5. Their final concentration in the reaction mix was 0.5 µM. The PCR cycling conditions were set at 95 °C for 20 s, 40 × [95 °C for 3 s, 60 °C for 20 s]. The melting curves were acquired by heating the samples to 95 °C for 1 s, cooling to 60 °C for 20 s and then slowly increasing the temperature from 60 °C to 95 °C at the rate of 0.15 °C · s^−1^, with a continuous measurement of the fluorescence. Amplification and melting curve data were generated and analyzed using the QuantStudioTM Design & Analysis Software v1.5.1 (ThermoFisher Scientific, Vantaa, Finland). For each gene, the efficiency was evaluated using serial dilutions of cDNA samples. The expression levels of the genes studied, normalized with the expression of the reference genes *ef1α* (Elongation factor 1-alpha) and *rpb2* (RNA Polymerase II Gene), were determined using the Relative Expression Software Tool (REST^®^, Qiagen, Courtaboeuf, France). Results were reported in the log2 value of the ratio treated condition with FER vs. control condition.

### 5.6. Expression of Results and Statistical Analyses

Results were presented as mean values ± standard deviation of three biological replications. Statistical analysis was performed with XLSTAT 2019 software (Addinsoft, Rennes, France). For all experimentations, ANOVA was used and differences between treaments were tested with Tukey post hoc test. The level of significance was set at *p* = 0.05.

## Figures and Tables

**Table 1:** Experimental half-maximal inhibitory concentration (IC_50_) ^1^ values of hydroxycinnamic acids against *F. avenaceum* I612 cultivated on solid Potato Dextrose Agar medium.

Phenolic Acids	CAF	FER	CHLO	COUM	SIN	SYR
IC_50_ (mM)	5.1	1.4	5.2	1.4	3.4	3.4

^1^ IC50 value of each phenolic acid was calculated using dose effect logistic model, on the basis of five tested concentrations and three replications for each concentration. CAF—caffeic acid, FER—ferulic acid, CHLO—chlorogenic acid, COUM—*p*-coumaric acid, SIN—sinapic acid, SYR—syringic acid.

**Table 2:** Effect of hydroxycinnamic acids at 0.5 mM on the production of enniatins by *F. avenaceum* I612 in *Fusarium* defined medium buffered at pH 3 and pH 6.

Phenolic Acid	pKa Value	Dry Fungal Biomass (mg)		Enniatins (A, A_1_, B, B_1_) Sum (µg · g^−1^ of Dry Biomass)	
		pH 3	pH 6	pH 3	pH 6
Control	−	46.0 ± 8.6	72.7 ± 1.4	1664.7 ± 175.6	1627.9 ± 179.5
CAF	4.62	72.6 ± 15.5 *	65.3 ± 1.1 *	1.23 ± 0.9 *	967.3 ± 176.5
FER	4.58	26.4 ± 2.9	79.6 ± 3.4 *	< LOD	621.0 ± 149.0 *
CHLO	2.66	67.1 ± 2.4 *	71.1 ± 3.4	31.6 ± 6.1 *	1872.3 ± 309.0
COUM	4.64	65.1 ± 3.4	68.5 ± 1.4	0.6 ± 0.2 *	441.5 ± 87.7 *
SIN	6.61	29.6 ± 0.1	76.5 ± 0.3	128.5 ± 33.3 *	1443.5 ± 309.6
SYR	3.90	31.8 ± 2.1	72.5 ± 1.4	336.0 ± 41.5 *	1662.1 ± 569.6

CAF—caffeic acid, FER—ferulic acid, CHLO—chlorogenic acid, COUM—*p*-coumaric acid, SIN—sinapic acid, SYR—syringic acid, LOD—limit of detection (0.01 µg · g^−1^). Data are means ± standard deviation using three biological replicates. Differences between control and tested phenolic acids were determined with multiple comparisons using Tukey test Asterix (*) indicates significant differences when compared with control (*p* < 0.05).

**Table 3:** Expression of *esyn1* and *kivr* genes in the ferulic acid supplemented culture relative to the control culture.

Medium	Gene	
	*esyn1*	*kivr*
FDM + FER/FDM	−3.4	−3.1
FDM pH 4 + FER/FDM pH 4	−11.50	−8.40
FDM pH 7 + FER/FDM pH 7	−0.75	−0.6

Values are expressed in log2 ratio scale ferulic acid condition vs. control condition, calculated using the REST software (Qiagen, Courtaboeuf, France). FDM—*Fusarium* defined media, FER—ferulic acid.

**Table 4:** Origin, isolation characteristics and toxigenic potential of the *F. avenaceum* strains used in this study.

Strain	Source	Host	Country and Year of Isolation	Sum of Enniatins ^1^ (µg · g^−1^)
FaLH27	Canadian collection of fungal cultures	Winter wheat	Canada, 2011	1152.4 ± 98.2
I612	INRAE/MycSA collection	Wheat	Scotland, 2010	2948.7 ± 21.0
I497	INRAE/MycSA collection	Soft Wheat	France, 2007	995.3 ± 13.3
I495	INRAE/MycSA collection	Soft Wheat	France, 2007	1360.3 ± 91.6
I112	INRAE/MycSA collection	Corn	France, 2001	105.2 ± 30.2
CBS 143.25	Centraal Bureau voor Shimmelkulturen, The Netherlands	N/A ^2^	N/A ^2^, 1926	31.3 ± 2.8

^1^ Sum of enniatin A, enniatin A1, enniatin B and enniatin B1 produced by *F. avenaceum* strains after 10 days of culture in *Fusarium* defined medium [48,49]. ^2^ N/A—Data not available.

**Table 5:** Targeted genes and associated primers used to amplify cDNA by real-time PCR.

Gene	Sequence Forwards (5′-3′)	Sequence Reverse (5′-3′)	Product (bp)	Accession No.
*kivr*	CGGAGACTAGACCACAGTAT	GCAAAGACGACAGAACTACA	151	JPYM01000010.1
*esyn1*	GAGTCCTCTCCCAAGTTC	AGTTGAAGACCACGAGAT	102	AF351597.2; AF351594.2; EF040582.1; Z18755.3;
*rpb2*	CCGAGGATCTCGAACTTTAC	CTTCCTCTTGTTCTCTCTTCTC	105	MH582082.1; MK560856.1 MK185026.1; MK185027.1, MK572784.1
*ef1α*	ACTTCCCCTCCAGGATGTCT	GTTACCACGTCGGATGTCCT	232	JQGD01000010.1
